# Supplementary material for: Content-rich biological network constructed by mining PubMed abstracts
Source: BMC Bioinformatics. 2004 Oct 8;5:147. doi: 10.1186/1471-2105-5-147 (PMC528731; doi:10.1186/1471-2105-5-147)
Supplement: Additional File 2 — The original results of the above study (non-essential files are deleted to keep the file size under the limit set by BMC bioinformatics). [file 1471-2105-5-147-S2.bz2 › chilibotAdditionalFile2/dip10/556ID10395669E10916/html/IFNAR2_IFNA1.html]

 


 **IFNAR2** and **IFNA1** 
  
Found 143 abstracts in PubMed, retrieved 10.  
 

 What does Google say? 
 PDF only 
| .edu only 

---

**Interactive relationship** (e.g. stimulation, inhibition, etc)

**Neutral relationship**- The interaction of IFNs with their heterodimeric type I IFN  [ **IFNA1** ]  receptor comprised of IFNAR1 and  **IFNAR2**  is a first step to inducing biological actions.  Ref: 12542398 Biochem J, 2003
- Liver biopsy specimens from 41 chronic hepatitis C patients subsequently treated with IFN  [ **IFNA1** ]  were immunostained with IFNAR1 and  **IFNAR2**  antibodies, and also analyzed for both receptor subunit mRNA levels, using competitive polymerase chain reaction.  Ref: 12699848 Hepatol Res< MedlineTA>Hepatol Res, 2003
- The concentration of type I interferon receptor  [ **IFNAR2** ]  IFN  [ **IFNA1** ]  Rc in the liver is a crucial factor in determining the efficacy of interferon IFN  [ **IFNA1** ]  therapy in patients with chronic hepatitis C.  Ref: 12518169 J Lab Clin Med, 2003
- These studies demonstrate the heavy concentration of IFNAR1 and  **IFNAR2**  in cells of the LE and superficial GE, which appear to be the main targets for IFN  [ **IFNA1** ]  tau.  Ref: 12193393 Biol Reprod, 2002

**Non-interactive relationship** (e.g. studied together, co-existance, homology, etc.)

- The focus of this study is to determine both mRNA and protein expression levels of the type I interferon IFN  [ **IFNA1** ]  receptor subunits, IFNalpha receptor IFNAR1 and IFNalpha beta receptor  **IFNAR2** , in the liver, and to assess which quantification method is most useful in predicting response to IFN  [ **IFNA1** ]  in chronic hepatitis C patients.  Ref: 12699848 Hepatol Res< MedlineTA>Hepatol Res, 2002
- RESULTS Single rectal administration of TNBS developed significant colitis in IFN  [ **IFNA1** ]  R  [ **IFNAR2** ]  mice and anti IFN  [ **IFNA1** ]  gamma mAb pretreated mice, as well as control wild type mice.  Ref: 12702051 J Gastroenterol Hepatol< MedlineTA>J Gastroenterol Hepatol, 2002
- To assess whether hepatitis C virus infection HCV affects type I interferon IFN  [ **IFNA1** ]  receptor expression, the expression levels of two subunits of this receptor, IFNAR1 and  **IFNAR2** , in peripheral blood mononuclear cells PBMCs were determined by flow cytometric assay in 68 anti HCV antibody positive patients 47 positive for HCV RNA and 21 negative for HCV RNA and 14 healthy controls.  Ref: 12787799 Hepatol Res< MedlineTA>Hepatol Res, 2002
- The structure of the  **IFNAR2**  IFN  [ **IFNA1** ]  binding ectodomain  **IFNAR2**  EC, the first helical cytokine receptor structure determined in solution, reveals the molecular basis for IFN  [ **IFNA1** ]  binding.  Ref: 12842042 Structure Camb , 2003
- Furthermore, adult Stat1, IFN  [ **IFNA1** ]  gamma, and IFN alpha  [ **IFNA1** ]  beta receptor IFNAR 2  [ **IFNAR2** ]  mice infected with rotavirus or vaccinated with VP6 vaccine and adjuvant were fully protected against rotavirus shedding following a subsequent challenge with rotavirus.  Ref: 12716489 J Interferon Cytokine Res, 2003
